# An Evaluation of HIV Elite Controller Definitions within a Large Seroconverter Cohort Collaboration

**DOI:** 10.1371/journal.pone.0086719

**Published:** 2014-01-28

**Authors:** Ashley D. Olson, Laurence Meyer, Maria Prins, Rodolphe Thiebaut, Deepti Gurdasani, Marguerite Guiguet, Marie-Laure Chaix, Pauli Amornkul, Abdel Babiker, Manjinder S. Sandhu, Kholoud Porter, for C. A. S. C. A. D. E. Collaboration in EuroCoord

**Affiliations:** 1 Medical Research Council Clinical Trials Unit at University College London, London, United Kingdom; 2 Institut National de la Santé et de la Recherche Médicale U1018, Université Paris-Sud, le Kremlin-Bicêtre, France; 3 Amsterdam Public Health Service, Amsterdam, Netherlands; 4 Institut National de la Santé et de la Recherche Médicale U897, Université Bordeaux Segalen, Bordeaux, France; 5 Wellcome Trust Sanger Institute, Hinxton, United Kingdom; 6 University of Cambridge, Cambridge, United Kingdom; 7 Institut National de la Santé et de la Recherche Médicale U943, Paris, France; 8 Université Pierre et Marie Curie S943, Paris, France; 9 Université Paris Descartes, EA 3620, Hôpital Necker-Enfants Malades, Paris, France; 10 International AIDS Vaccine Initiative, San Francisco, California, United States of America; Infectious Disease Service, United States of America

## Abstract

**Background:**

Understanding the mechanisms underlying viral control is highly relevant to vaccine studies and elite control (EC) of HIV infection. Although numerous definitions of EC exist, it is not clear which, if any, best identify this rare phenotype.

**Methods:**

We assessed a number of EC definitions used in the literature using CASCADE data of 25,692 HIV seroconverters. We estimated proportions maintaining EC of total ART-naïve follow-up time, and disease progression, comparing to non-EC. We also examined HIV-RNA and CD4 values and CD4 slope during EC and beyond (while ART naïve).

**Results:**

Most definitions classify ∼1% as ECs with median HIV-RNA 43–903 copies/ml and median CD4>500 cells/mm^3^. Beyond EC status, median HIV-RNA levels remained low, although often detectable, and CD4 values high but with strong evidence of decline for all definitions. Median % ART-naïve time as EC was ≥92% although overlap between definitions was low. EC definitions with consecutive HIV-RNA measurements <75 copies/ml with follow-up≥ six months, or with 90% of measurements <400 copies/ml over ≥10 year follow-up preformed best overall. Individuals thus defined were less likely to progress to endpoint (hazard ratios ranged from 12.5–19.0 for non-ECs compared to ECs).

**Conclusions:**

ECs are rare, less likely to progress to clinical disease, but may eventually lose control. We suggest definitions requiring individuals to have consecutive undetectable HIV-RNA measurements for ≥ six months or otherwise with >90% of measurements <400 copies/ml over ≥10 years be used to define this phenotype.

## Introduction

HIV is typically characterised by a period of viral replication and CD4 cell decline leading to AIDS and death in the absence of antiretroviral therapy (ART). [1] Differences in the evolution of both markers over time, however, result in large variations in disease progression among HIV-positive individuals. [2], [3] The long-term non-progressor (LTNP) phenotype was initially described to characterise individuals who experienced slow disease progression and stable CD4 counts over a number of years. [4], [5] With the introduction of HIV-RNA assays in the mid-1990s, research shifted to focus on mechanisms which lead to control of viral replication [6].

A small proportion of individuals have been described who are able to suppress viral replication to undetectable levels for extended periods of time without use of ART, delaying the onset of AIDS. [7], [8] Many terms are used in the literature for such individuals, with the most common being elite controllers (EC). [8] Mechanisms of EC remain unclear, although it is now believed that host response, including CD4 and CD8 T cell-specific immune response, [9], [10] as well as HLA Class I alleles, [11] are likely to be the main mechanisms of control, rather than infection by defective virus, as initially postulated. [12] Whatever the mechanisms, a study of this group of individuals gives potential for the development of new treatment strategies, can guide research on HIV vaccines,[13]–[16] and provide models for a functional cure of HIV [17], [18].

Since the publication of initial definitions of EC, as was the case with the long-term non-progressor (LTNP) phenotype, many more definitions have been proposed; presumably to arrive at one definition which best defines true EC. There are currently numerous definitions, each of which differs by the follow-up time required and the number and threshold of undetectable HIV-RNA measurements.[6]–[8], [15], [16], [19]–[23] It is not known which, if any, best characterise this rare phenotype, however. This is important to ensure that any difference between elite controllers and non-controllers can be attributed to the phenotype itself. An assessment of the relative merits of each definition has never been undertaken or a comparison between them preformed.

The CASCADE (Concerted Action on SeroConversion on AIDS and Death in Europe) Collaboration, of HIV-positive individuals followed-up since HIV seroconversion, offers a unique opportunity to assess the ability of these definitions to capture EC. Using data from CASCADE, we aimed to evaluate a number of commonly-used definitions to estimate prevalence of EC and associated factors, proportion of total follow-up time spent as elite, and to describe CD4 and HIV-RNA values during EC and beyond the EC period.

The work provides the basis for choosing a definition appropriate to the objectives of future research on this rare phenotype.

## Materials and Methods

### Study Population

We used pooled data from the CASCADE September 2011 data release in EuroCoord (www.EuroCoord.net), which consists of 25,629 seroconverters from 28 cohorts across Europe, Canada, Australia and sub-Saharan Africa. [24] Date of seroconversion is estimated by various methods, most commonly as the midpoint between the last documented HIV negative and the first positive HIV antibody test dates with an interval of <3 years between the two test dates (85%). For the remainder, date of seroconversion was estimated through laboratory evidence of seroconversion (PCR positivity in the absence of HIV antibodies or antigen positivity with fewer than four bands on Western blot) (13%), or as the date of a seroconversion illness (2%) with both an earlier documented negative and a later positive HIV test not more than 3 years apart. Anonymized data for the CASCADE collaboration are collected and stored at the Medical Research Council Clinical Trials Unit at University College London. Access is available to bona fide researchers through submission of a proposal to the Steering Committee which is reviewed by CASCADE investigators.

### Elite Control Definitions

We undertook a systematic review of the literature, which is described elsewhere [25]. Briefly, we searched for terms previously used to describe control of HIV infection including “Long term non-progressors”, “LTNP”, “elite controller”, “elite control”, “viral controller” and “viral control” and evaluated 10 EC definitions (Table 1).[6]–[8], [15], [16], [19]–[23] The list of definitions included in this paper is not intended to be exhaustive; rather it is representative of the spectrum by which the elite control phenotype is defined in the literature. Three definitions were most commonly used, all requiring HIV positive individuals to meet the following criteria while ART-naive and AIDS-free: 1) Definition E, used by the International HIV Controllers Consortium, of individuals who maintain HIV-RNA levels below 75 copies/mL for at least 1 year, [8] 2) Definition F, an adaptation of definition E allowing no previous HIV-RNA levels >1000 copies/ml, [6] and 3) Definition J, initially proposed by the ANRS, of individuals known to be HIV positive for ≥10 years with ≥2 HIV-RNA measurements, ≥90% of which were required to be <400 copies/ml [7].

**Table 1:** 10 definitions of elite control from the literature applied to the CASCADE dataset; all require individuals to be AIDS-free and ART-naïve.

*Definition*	
**A**	HIV-positive ≥6 months, with ≥2 consecutive HIV-RNA <75 copies/ml [22]
**B**	HIV-positive ≥1 year, with ≥1 HIV-RNA <50 copies/ml [16]
**C**	HIV-positive ≥1 year, with ≥1 HIV-RNA <75 copies/ml [15]
**D**	HIV-positive ≥1 year, with ≥3 HIV-RNA <2000 copies/ml [21]
**E**	HIV-positive ≥1 year, with ≥3 consecutive HIV-RNA <75 copies/ml spanning ≥12 months [8]
**F**	HIV-positive ≥1 year, with ≥3 consecutive HIV-RNA <75 copies/ml spanning ≥12 months with no previous blips ≥1000 copies/ml [6]
**G**	HIV-positive ≥2 years, with ≥2 HIV-RNA <75 copies/ml [19]
**H**	HIV-positive ≥5 years, with ≥5 consecutive HIV-RNA <500 copies/ml [23]
**I**	HIV-positive ≥10 years, with all measured HIV-RNA <50 copies/ml [20]
**J**	HIV-positive ≥10 years, with ≥90% of HIV-RNA (≥2 HIV-RNA ever) <400 copies/ml [7]

### Statistical Methods

We identified three groups of individuals for each definition: those who fulfilled it, those who did not, and those whose EC status could not be determined, e.g. because insufficient follow-up or ART-naive HIV-RNA measurements were not available. For each definition we estimated the proportion of EC excluding individuals whose EC status was unknown from the denominator. Because there were large numbers with unknown status, we also estimated proportion of EC by assuming them to be non-EC and including them in the denominator, thus providing minimum proportion estimates.

For each definition we estimated the proportion of time they remained as EC by considering all available ART-naive follow-up. To estimate the effect of EC status on disease progression we restricted entry to the risk set at 10 years post seroconversion, as this was the longest duration of follow-up required by all definitions considered, and used multivariable time dependent Cox proportional hazards models to estimate the hazard ratio for a composite endpoint of AIDS, death (all cause), ART initiation or CD4<350 cells/mm^3^ comparing non-ECs and unknowns to ECs. We formally tested differences in hazard ratios between the definitions by using 1000 bootstrap replicates. Definition I was excluded from the bootstrap analysis as there were few follow-up measurements among a small number of elite controllers providing unstable estimates.

We described median and interquartile ranges (IQR) of HIV-RNA and CD4 levels, based on median individual values, while classified as EC and during total ART-naïve follow-up time. CD4 slopes were estimated using a linear mixed model on the square root scale, while classified as an elite controller, and also during total ART-naïve follow-up time.

## Results

Data from 28 cohorts of 25,692 individuals formed the base from which sub-populations of EC and non-EC were drawn according to each definition. Median (IQR) year of HIV seroconversion was 1999 (1992, 2005) and median age at seroconversion 31 years (25, 37). HIV risk groups were MSM (55%), MSW (26%) or IDU (14%), and the majority were male (78%).

### Proportion Classified as EC and Patient Characteristics

The proportion classified as EC by each definition was 0.15–7.70% and did not necessarily reflect the length of follow-up required by the definition (**Table 2**). While variations in age, sex and risk group were observed for each definition, no consistent differences were observed across definitions.

**Table 2:** Number of elite controllers (EC), their proportion, and demographic characteristics applying the CASCADE dataset to 10 definitions of EC found in the literature.

*Def*.	*EC* *(n)*	*Non-EC* *(n)*	*Unknown†* *(n)*	*EC* *Proportion* *Best* *Estimate‡* *n (%)*	*EC* *Proportion* *Minimum* *Estimate 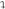 * *n (%)*	*Seroconversion* *Age (Median)*		*Male* *(%)*		*MSM 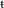 * *(%)*		*IDU 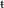 * *(%)*		*MSW 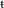 * *(%)*	
						*ECs*	*Non* *ECs*	*ECs*	*Non* *ECs*	*ECs*	*Non* *ECs*	*ECs*	*Non* *ECs*	*ECs*	*Non* *ECs*
**A** ŧ	282	20951	4396	1.33	1.10	32	31	74	78	53	57	11	11	32	27
**B** ŧ	495	19568	5566	2.47	1.93	32	31	79	78	59	56	15	11	23	28
**C** ŧ	827	19236	5566	4.12	3.23	32	31	74	78	54	56	11	11	31	27
**D** ŧ	1416	16964	7249	7.70	5.52	30	31	67	79	49	58	11	10	36	27
**E** ŧ	174	17160	8295	1.00	0.68	33	31	75	78	52	57	13	10	30	28
**F** ŧ	95	17239	8295	0.55	0.37	32	31	63	78	37	57	18	10	38	28
**G** ŧ	392	16891	8346	2.27	1.53	32	31	76	77	55	56	12	11	30	28
**H** ŧ	146	10899	14584	1.32	0.57	31	30	74	77	47	55	18	13	29	27
**I** ŧ	10	6694	18925	0.15	0.04	26	29	80	77	30	53	60	18	0	25
**J** ŧ	47	6554	19028	0.71	0.18	31	29	74	77	34	53	28	17	30	25

† Individuals in the cohort without adequate follow-up or number of HIV-RNA measurements to classify them as EC or non-EC.

‡ Based on number of seroconverters whose EC status could be determined.

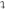
HIV risk groups: MSM: Men who have sex with men; IDU: Injection drug users; MSW: Heterosexual contact.

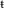
Assuming all individuals with unknown EC status are non-EC.

ŧ **A**: HIV-positive ≥6 months, with ≥2 consecutive HIV-RNA <75 copies/ml; **B**: HIV- positive ≥1 year, with ≥1 HIV-RNA <50 copies/ml, **C**: HIV- positive ≥1 year, with ≥1 HIV-RNA <75 copies/ml, **D**: HIV- positive ≥1 year, with ≥3 HIV-RNA <2000 copies/ml, **E**: HIV- positive ≥1 year, with ≥3 consecutive HIV-RNA <75 copies/ml spanning ≥12 months **F**: HIV- positive ≥1 year, with ≥3 consecutive HIV-RNA <75 copies/ml spanning ≥12 months with no previous blips ≥1000 copies/ml, **G**:HIV- positive ≥2 years, with ≥2 HIV-RNA <75 copies/ml, **H**: HIV- positive ≥5 years, with ≥5 consecutive HIV-RNA <500 copies/ml, **I**: HIV- positive ≥10 years, with all measured HIV-RNA <50 copies/ml, **J**: HIV-positive ≥10 years, with ≥90% of HIV-RNA (≥2 HIV-RNA ever) <400 copies/ml.

The number of individuals fulfilling two definitions was generally low with 33% of individuals overlapping by <30% with another definition (**Table 3**).

**Table 3:** Two-way overlap of 10 definitions of elite control found in the literature applied to the CASCADE dataset.

*Def.*	*A, n (%)*	*B, n (%)*	*C, n (%)*	*D, n (%)*	*E, n (%)*	*F, n (%)*	*G, n (%)*	*H, n (%)*	*I, n (%)*	*J, n (%)*	*Total*
**A** ŧ	–	195 (39)	279 (34)	275 (19)	174 (100)	95 (100)	250 (64)	113 (77)	4 (40)	35 (74)	282
**B** ŧ	195 (69)	–	495 (60)	341 (24)	119 (68)	45 (47)	286 (73)	94 (64)	10 (100)	36 (77)	495
**C** ŧ	279 (99)	495 (100)	–	542 (38)	174 (100)	95 (100)	392 (100)	133 (91)	10 (100)	42 (89)	827
**D** ŧ	275 (98)	341 (69)	542 (66)	–	174 (100)	95 (100)	354 (90)	146 (100)	4 (40)	41 (87)	1416
**E** ŧ	174 (62)	119 (24)	174 (21)	174 (12)	–	95 (100)	165 (42)	95 (65)	3 (30)	31 (66)	174
**F** ŧ	95 (34)	45 (9)	95 (11)	95 (7)	95 (55)	–	91 (23)	53 (36)	3 (30)	25 (53)	95
**G** ŧ	250 (89)	286 (58)	392 (47)	354 (25)	165 (95)	91 (96)	–	125 (86)	6 (60)	41 (87)	392
**H** ŧ	113 (40)	94 (19)	133 (16)	146 (10)	95 (55)	53 (56)	125 (32)	–	3 (30)	29 (62)	146
**I** ŧ	4 (1)	10 (2)	10 (1)	4 (0)	3 (2)	3 (3)	6 (2)	3 (2)	–	6 (13)	10
**J** ŧ	35 (12)	36 (7)	42 (5)	41 (3)	31 (18)	25 (26)	41 (10)	29 (20)	6 (60)	–	47
**Total**	282	495	827	1416	174	95	392	146	10	47	

Example: 95 seroconverters were classified as EC by definition F of whom 25 (26%) were classified as EC by definition J. Conversely, of 47 seroconverters classified as EC by definition J, 25 (53%) were classified as EC by definition F.

ŧ **A**: HIV-positive ≥6 months, with ≥2 consecutive HIV-RNA <75 copies/ml; **B**: HIV- positive ≥1 year, with ≥1 HIV-RNA <50 copies/ml, **C**: HIV- positive ≥1 year, with ≥1 HIV-RNA <75 copies/ml, **D**: HIV- positive ≥1 year, with ≥3 HIV-RNA <2000 copies/ml, **E**: HIV- positive ≥1 year, with ≥3 consecutive HIV-RNA <75 copies/ml spanning ≥12 months **F**: HIV- positive ≥1 year, with ≥3 consecutive HIV-RNA <75 copies/ml spanning ≥12 months with no previous blips ≥1000 copies/ml, **G**:HIV- positive ≥2 years, with ≥2 HIV-RNA <75 copies/ml, **H**: HIV- positive ≥5 years, with ≥5 consecutive HIV-RNA <500 copies/ml, **I**: HIV- positive ≥10 years, with all measured HIV-RNA <50 copies/ml, **J**: HIV-positive ≥10 years, with ≥90% of HIV-RNA (≥2 HIV-RNA ever) <400 copies/ml.

### Total Time Spent as EC

The risk of composite endpoint was consistently significantly higher for non-EC/unknown compared to ECs for all definitions, with hazard ratios ranging from 2.9–19.0 and being greatest for definitions A, E, F and J (**Table 4**). 1000 bootstrap replicates confirmed the superiority of A, E and F (α = 0.05) above definitions B, C, D, G and H, the former 3 definitions not being statistically different from each other. Definition J was not statistically superior to any other definition.

**Table 4:** Estimated hazard ratios comparing non-elite controllers (EC) and unknown to EC for time from estimated HIV seroconversion to a composite endpoint of AIDS, Death, ART, or CD4<350 cells/mm^3^ restricting entry to the risk set at 10 years post seroconversion using the CASCADE dataset applied to 10 definitions of EC found in the literature.

*Def.*	*EC evaluated (experiencing* *composite endpoint) n (n)* ††	*HR for time to composite* *endpoint* † *(95% CI)*	*% (IQR) ART-naïve follow-up* *time classified as EC*
**A** ŧ	46 (4)	12.5 (4.7, 33.6)	100 (78–100)
**B** ŧ	53 (11)	4.6 (2.5, 8.3)‡	100 (78–100)
**C** ŧ	86 (18)	4.8 (3.0, 7.7)‡	99 (72–100)
**D** ŧ	134 (35)	4.0 (2.8, 5.7)‡	97 (71–100)
**E** ŧ	36 (2)	19.0 (4.7, 76.4)	100 (78–100)
**F** ŧ	26 (5)	15.3 (3.8, 61.3)	92 (66–100)
**G** ŧ	60 (9)	7.5 (3.9, 14.5)‡	100 (86–100)
**H** ŧ	56 (22)	2.9 (1.9, 4.4)‡	100 (75–100)
**I** ŧ	4 (1)	3.4 (0.5, 24.0)	100 (100–100)
**J** ŧ	35 (3)	13.2 (4.2, 41.3)	100 (98–100)

†† Number of Elites making it to 10 years follow up without experiencing composite endpoint and number subsequently experiencing composite endpoint.

† Hazard ratios comparing ECs to Non-ECs (including those with unknown EC status) allowing for late entry at 10 years. For each definition, p-values were obtained from unadjusted log-rank test for time to composite endpoint and were all highly significant p<0.001.

‡ Statistically different HRs compared to definition E, F, and A from 1000 bootstrap replicates. No definitions were statistically different from definition J at α = 0.05.

ŧ **A**: HIV-positive ≥6 months, with ≥2 consecutive HIV-RNA <75 copies/ml; **B**: HIV- positive ≥1 year, with ≥1 HIV-RNA <50 copies/ml, **C**: HIV- positive ≥1 year, with ≥1 HIV-RNA <75 copies/ml, **D**: HIV- positive ≥1 year, with ≥3 HIV-RNA <2000 copies/ml, **E**: HIV- positive ≥1 year, with ≥3 consecutive HIV-RNA <75 copies/ml spanning ≥12 months **F**: HIV- positive ≥1 year, with ≥3 consecutive HIV-RNA <75 copies/ml spanning ≥12 months with no previous blips ≥1000 copies/ml, **G**:HIV- positive ≥2 years, with ≥2 HIV-RNA <75 copies/ml, **H**: HIV- positive ≥5 years, with ≥5 consecutive HIV-RNA <500 copies/ml, **I**: HIV- positive ≥10 years, with all measured HIV-RNA <50 copies/ml, **J**: HIV-positive ≥10 years, with ≥90% of HIV-RNA (≥2 HIV-RNA ever) <400 copies/ml.

**Figure 1:**
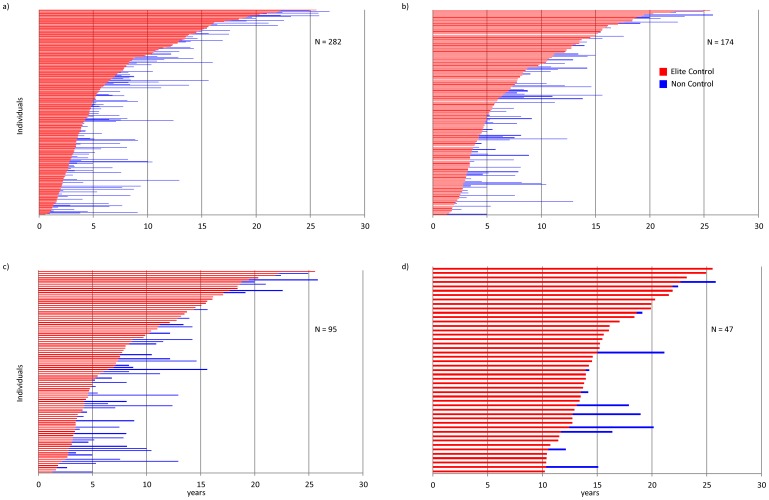
Total ART-naïve follow-up time spent as an elite controller for 4 of the best performing definitions: a) A^ ŧ^, b) E^ ŧ^, c) F^ ŧ^,and d) J^ ŧ^ using the CSCADE dataset. ^ŧ^ **A:** HIV-positive ≥6 months, with ≥2 consecutive HIV-RNA <75 copies/ml; **E:** HIV- positive ≥1 year, with ≥3 consecutive HIV-RNA <75 copies/ml spanning ≥12 months **F:** HIV- positive ≥1 year, with ≥3 consecutive HIV-RNA <75 copies/ml spanning ≥12 months with no previous blips ≥1000 copies/ml, J: HIV- positive ≥10 years, with ≥90% of HIV-RNA (≥2 HIV-RNA ever) <400 copies/ml.

Considering all available ART-naïve follow-up from seroconversion, the proportion of time spent as EC, according to each definition, was remarkably high with median follow-up≥92% for all definitions, although 25% of EC, according to definitions C, D and F, spent ≤72% of their ART-naïve follow-up time as EC (**Table 4**). Figure 1 illustrates total ART-naive follow-up for all individuals classified as EC by definitions A, E, F, and J.

### HIV-RNA and CD4 Values during EC Status

Median HIV-RNA during the time of EC was generally low for all definitions varying from 35–903 copies/ml and median CD4 levels high at >500 cells/mm^3^ (**Table 5**). There was strong evidence of CD4 loss during this EC period, however, for at least 5 of the 10 definitions considered. For the remaining definitions, slopes were either level (no strong evidence of CD4 loss) or otherwise with a statistically significant positive slope. Such positive slopes are likely due to short follow-up, chance, or possibly informative censoring, as follow-up is censored for those with a negative slope once ART is initiated [26]. Median HIV-RNA, CD4 values and CD4 slopes during EC status excluding counts within 6 months of seroconversion showed similar results (data not shown).

**Table 5:** HIV-RNA and CD4 values and estimated CD4 slope during elite control (EC), and throughout ART-naïve follow-up using the CASCADE dataset applied to 10 definitions of EC found in the literature.

*Def.*	*During Elite Control*			*During ART-naïve follow-up*		
	*HIV-RNA value*	*CD4 Value*	*CD4 slope* † *(95% CI)*	*HIV-RNA value*	*CD4 Value*	*CD4 slope* † *(95% CI)*
**A^ŧ^**	50 (35, 276)	675 (454, 877)	0.04 (0.01, 0.08)	66 (35, 495)	654 (441, 840)	−0.09 (−0.12, −0.06)‡
**B^ŧ^**	425 (35, 11641)	573 (409, 792)	−0.16 (−0.19, −0.12)‡	1043 (89, 13000)	548 (404, 751)	−0.28 (−0.30, −0.25)‡
**C^ŧ^**	354 (50, 8700)	596 (427, 796)	−0.18 (−0.21, −0.15)‡	660 (75, 11066)	567 (415, 764)	−0.31 (−0.34, −0.29)‡
**D^ŧ^**	903 (287, 1863)	615 (478, 789)	−0.27 (−0.29, −0.25)‡	1274 (370, 3304)	590 (451, 756)	−0.43 (−0.45, −0.41)‡
**E^ŧ^**	50 (35, 81)	699 (528, 922)	0.06 (0.01, 0.10)	50 (35, 165)	681 (527, 909)	−0.06 (−0.10, −0.02)
**F^ŧ^**	50 (35, 50)	839 (654, 1070)	0.05 (−0.00, 0.11)	50 (35, 77)	796 (629, 1020)	−0.08 (−0.13, −0.03)
**G^ŧ^**	113 (49, 1197)	644 (439, 824)	−0.06 (−0.09, −0.03)‡	176 (50, 2160)	625 (438, 806)	−0.15 (−0.18, −0.13)‡
**H^ŧ^**	76 (35, 283)	697 (541, 879)	−0.09 (−0.12, −0.05)‡	89 (35, 356)	687 (530, 879)	−0.23 (−0.26, −0.20)‡
**I^ŧ^**	35 (1, 35)	583 (575, 905)	−0.07 (−0.24, 0.09)	35 (1, 35)	583 (575, 905)	−0.05 (−0.21, 0.11)
**J^ŧ^**	50 (35, 127)	783 (628, 970)	−0.03 (−0.09, 0.02)	50 (35, 169)	740 (583, 970)	−0.11 (−0.16, −0.06)‡

Note- all values unless otherwise stated are median (IQR).

† CD4 slope modelled on the square root scale with linear mixed models, specific p-values for CD4 slope and median number of CD4 measurements are presented in Table S1.

‡ CD4 slope highly significant p<0.001.

Ŧ Number of total CD4 and HIV-RNA measurements **^ŧ^ A**: HIV-positive ≥6 months, with ≥2 consecutive HIV-RNA <75 copies/ml; **B**: HIV- positive ≥1 year, with ≥1 HIV-RNA <50 copies/ml, **C**: HIV- positive ≥1 year, with ≥1 HIV-RNA <75 copies/ml, **D**: HIV- positive ≥1 year, with ≥3 HIV-RNA <2000 copies/ml, **E**: HIV- positive ≥1 year, with ≥3 consecutive HIV-RNA <75 copies/ml spanning ≥12 months **F**: HIV- positive ≥1 year, with ≥3 consecutive HIV-RNA <75 copies/ml spanning ≥12 months with no previous blips ≥1000 copies/ml, **G**:HIV- positive ≥2 years, with ≥2 HIV-RNA <75 copies/ml, **H**: HIV- positive ≥5 years, with ≥5 consecutive HIV-RNA <500 copies/ml, **I**: HIV- positive ≥10 years, with all measured HIV-RNA <50 copies/ml, **J**: HIV-positive ≥10 years, with ≥90% of HIV-RNA (≥2 HIV-RNA ever) <400 copies/ml.

### HIV-RNA and CD4 Values during Total ART-naïve Follow-up

As expected, throughout available ART-naïve follow-up, median HIV-RNA values were generally higher, and CD4 counts lower than those considering only the time spent as EC. Nevertheless, median HIV-RNA throughout ART-naïve follow-up was low, <200 copies/ml for most definitions, and median CD4 values were >500 cells/mm^3^ for all definitions (**Table 5**).

Of note, however, CD4 slopes during total ART-naïve follow-up were significantly negative (α = 0.05) for all but one definition. HIV-RNA and CD4 values and CD4 slopes showed consistent results when CD4 values within 6 months of seroconversion were excluded (data not shown).

## Discussion

Using the large size of the CASCADE dataset we were able to provide reliable estimates of the proportion likely to be elite controllers in an HIV-positive population. Our findings confirm that, by whichever definition, elite control is a rare phenotype likely to comprise around 1% of individuals. This is in line with estimates reported by others [7], [8], [23] although it should be noted that the choice of denominator may distort the proportion (for example, considering all individuals regardless of their length of follow-up or HIV infection duration will tend to under-estimate this proportion of ECs). Interestingly, we also find evidence that ECs may eventually lose control of viraemia.

Definitions A, E, F and J which require low consecutive or a high proportion of low HIV-RNA measurements are best at capturing individuals with the slowest disease progression. When restricting the dataset to those with 10 years of follow-up, definitions A, E and F and J demonstrated the lowest hazard of AIDS, Death, ART or CD4<350 cells/mm^3^ compared to definitions with single measurements or higher levels of viremia. Definition J, with the longest follow-up of 10 years was not significantly different from all other definitions, although this is likely due to low numbers of individuals classified by this definition.

The proportion classified as EC varied according to each definition, with definition D, requiring an HIV-RNA threshold of <2000 copies/ml, classifying the greatest proportion as EC. This definition performed particularly poorly overall with the highest median HIV-RNA and fastest CD4 cell loss while classified as EC and during ART naïve follow-up. Given that the lower limit for available assays has been less than 1000 copies/ml for at least 10 years, inclusion of 2000 copies/ml limit is justifiably termed “viral controllers” rather than EC. The requirement of only one HIV-RNA measurement below a certain threshold (B and C) also resulted in relatively high proportions of EC (2.47% and 4.12%, respectively), agreeing with studies previously reporting proportions of individuals with ≥1 HIV-RNA undetectable [27], [28], and suggesting that one undetectable measurement is insufficient in defining EC status. HIV-RNA values were relatively high during EC period for both definitions with the upper quartile experiencing HIV-RNA values >8000 copies/ml. Similarly, even while classified as EC, CD4 cell counts were significantly declining. Thus, the use of at least two HIV-RNA counts results in more robust measures of stable viraemia and thus captures individuals with lower risk of disease progression.

In contrast, definitions I, J, E and F had the lowest proportion classified as EC varying between 0.15–1.00%. While this is expected for definitions that required ten years of follow-up (I and J), definition E and F, requiring only one year follow-up seemed to capture an equally rare group. The proportion of EC according to definition F was much lower than for definition E, indicating that inclusion of a criterion and threshold for viral blips, as defined by spikes in viral replication and subsequently maintaining control, does impact the group of individuals captured by the definition. This is consistent with studies suggesting that blips are not uncommon among elite controllers. [7], [29] In addition to selecting for a rare group, definitions E, F and J also selected groups with the lowest risk for the composite outcome, suggesting that these definitions capture a rare and extreme group on the clinical spectrum of HIV infection. Unsurprisingly, definition I led to the classification of the smallest proportion (0.15%) of EC. This is the most stringent definition as all HIV-RNA measurements needed to have been quantified by assays with a lower limit of detection <50 copies/ml for ≥10 years. The denominator from which this population has been drawn is, by definition, limited to the most recent period when routinely used assays had such low detection limits. Median HIV-RNA and CD4 values for those classified as EC by this definition are based on few measurements and are, therefore, unreliable.

Interestingly, a greater duration of follow-up did not necessarily lead to a more clinically-extreme group of individuals being identified, as definition E and J performed similarly, in spite of E requiring only 1 year of follow-up. There were 16 individuals classified by J but not by E. Six of these 16 were known to have naïve HIV-RNAs between 75 and 400 copies/ml, while HIV-RNA for the remaining 10 individuals were measured using assays with a 400 copies/ml lower detection threshold. Of 143 individuals classified as EC by E but not by J, 113 had <10 years of naïve follow-up. It may, therefore, be that overlap could be greater had all individuals been measured for the same duration with similar assays. In addition, median CD4 counts, HIV-RNA levels and CD4 slopes during the ART-naïve period were also similar for these definitions. This observation may have important implications for the design of future studies, as it seems to suggest that stringent definitions requiring only one year of follow-up, with consecutive undetectable HIV-RNA measurements, can identify an extreme group comparable to that identified by definitions requiring much longer follow-up and higher HIV-RNA threshold. There is, therefore, potential to sampling of participants in such studies from a much wider cohort of individuals.

It is important to note that, despite the fairly low levels of viraemia in individuals classified as EC over extended periods, there was strong evidence of CD4 cell loss, the exception being those classified as EC by definition I, which was based on relatively few measurements. Whether true LTNP status exists remains unknown [30], [31]. Our findings lead us to conclude, however, that this is unlikely among elite controllers.

Our study has several strengths. First, the large size of our cohort allows us to make reliable comparisons of different definitions of such a rare group of individuals. Second, the availability of seroconversion information allows for a meaningful assessment of time to clinical outcomes to be made. Finally, and most importantly, until now, examination of CD4 and HIV-RNA changes in studies has been restricted to the period in which HIV-positive individuals fulfil the respective definition. [29], [32], [33] Due to the detailed information available to us, we were able to study evolution of both these markers over an extended period of follow-up, beyond the duration of EC as defined.

The main limitation to this study is for each definition, the number of individuals with unknown EC classification varied which could have introduced bias in proportion estimates. This is most evident in definition J, requiring >2 HIV-RNA measurements and at least 10 years of follow-up with >19,000 individuals with either insufficient follow-up or number of HIV-RNA measurements. To examine the impact of missing data on this, for each definition we classified individuals with inadequate information (insufficient number of HIV-RNA measurements or follow-up requirements specified by the definition) as EC and then as non-EC. The proportion of EC; however, may theoretically range from 0.04, if all unknowns are classified as non-EC, to 74%, if all unknowns are classified as EC (data not shown) indicating the difficulties with estimating the true proportion of this group in the presence of missing data. In spite of these possible limitations, our study highlights important differences captured by different EC definitions.

In conclusion, identification of a rare and extreme group may be possible even with definitions requiring a relatively short period of follow-up. We have shown that definitions requiring 6 months or more of follow-up with consecutive measurements requiring HIV-RNA ≤75 copies/ml preform just as well as definitions requiring ≥10 years follow-up with HIV-RNA measured using assays with a higher detection limit. Although Definition E preforms best overall in terms of percent classified, time to composite endpoint, percent of naïve follow-up time spent as EC, HIV-RNA, and CD4 decline, definition A (2 consecutive HIV-RNA <75 copies/ml over 6 months), F (similar to E, but not allowing for blips above 1000 copies/ml) and J (10 years of follow-up with 90% HIV-RNA <400 copies/ml) also have their merits. It is unlikely, however, that elite control is an indefinite state, and that the few HIV-positive individuals who spontaneously control HIV replication may eventually need treatment or develop AIDS given the on-going, albeit slow, CD4 cell loss. However, ECs are much less likely to progress to clinical disease compared with non-ECs, and a better understanding of the mechanisms that lead to such control over extended periods may lead to new therapeutic strategies or the development of HIV vaccines.

## Supporting Information

Table S1 **Number of HIV-RNA and CD4 measurements during elite control and ART naïve follow-up, time from SC to first HIV-RNA and number of HIV-RNA measurements within 6 months of HIV positive test date using the CASCADE dataset from 10 definitions found in the literature.** Note- all values unless otherwise stated are median (IQR) †CD4 slope modelled on the square root scale with linear mixed models ^ŧ^ **A**: HIV-positive ≥6 months, with ≥2 consecutive HIV-RNA <75 copies/ml; **B**: HIV- positive ≥1 year, with ≥1 HIV-RNA <50 copies/ml, **C**: HIV- positive ≥1 year, with ≥1 HIV-RNA <75 copies/ml, **D**: HIV- positive ≥1 year, with ≥3 HIV-RNA <2000 copies/ml, **E**: HIV- positive ≥1 year, with ≥3 consecutive HIV-RNA <75 copies/ml spanning ≥12 months **F**: HIV- positive ≥1 year, with ≥3 consecutive HIV-RNA <75 copies/ml spanning ≥12 months with no previous blips ≥1000 copies/ml, **G**:HIV- positive ≥2 years, with ≥2 HIV-RNA <75 copies/ml, **H**: HIV- positive ≥5 years, with ≥5 consecutive HIV-RNA <500 copies/ml, **I**: HIV- positive ≥10 years, with all measured HIV-RNA <50 copies/ml, **J**: HIV-positive ≥10 years, with ≥90% of HIV-RNA (≥2 HIV-RNA ever) <400 copies/ml.
(DOCX)
