# Supplementary material for: An Evaluation of HIV Elite Controller Definitions within a Large Seroconverter Cohort Collaboration
Source: PLoS One. 2014 Jan 28;9(1):e86719. doi: 10.1371/journal.pone.0086719 (PMC3904947; doi:10.1371/journal.pone.0086719)
Supplement: Table S1 — Number of HIV-RNA and CD4 measurements during elite control and ART naïve follow-up, time from SC to first HIV-RNA and number of HIV-RNA measurements within 6 months of HIV positive test date using the CASCADE dataset from 10 definitions found in the literature. Note- all values unless otherwise stated are median (IQR) †CD4 slope modelled on the square root scale with linear mixed models ŧ A: HIV-positive ≥6 months, with ≥2 consecutive HIV-RNA <75 copies/ml; B: HIV- positive ≥1 year, with ≥1 HIV-RNA <50 copies/ml, C: HIV- positive ≥1 year, with ≥1 HIV-RNA <75 copies/ml, D: HIV- positive ≥1 year, with ≥3 HIV-RNA <2000 copies/ml, E: HIV- positive ≥1 year, with ≥3 consecutive HIV-RNA <75 copies/ml spanning ≥12 months F: HIV- positive ≥1 year, with ≥3 consecutive HIV-RNA <75 copies/ml spanning ≥12 months with no previous blips ≥1000 copies/ml, G:HIV- positive ≥2 years, with ≥2 HIV-RNA <75 copies/ml, H: HIV- positive ≥5 years, with ≥5 consecutive HIV-RNA <500 copies/ml, I: HIV- positive ≥10 years, with all measured HIV-RNA <50 copies/ml, J: HIV-positive ≥10 years, with ≥90% of HIV-RNA (≥2 HIV-RNA ever) <400 copies/ml. (DOCX) [file pone.0086719.s001.docx]

| ***Def.*** | ***During Elite Control*** |  |  |  | ***During ART-naïve follow-up*** |  |  | ***Time (months) from SC to first HIV-RNA*** | ***# HIV-RNA measurements within 6 months of HIV positive*** |
| --- | --- | --- | --- | --- | --- | --- | --- | --- | --- |
|  | ***# HIV-RNA measured*** | ***# CD4 measured*** | ***CD4 slope***† ***p-value*** |  | ***# HIV-RNA measured*** | ***# CD4 measured*** | ***CD4 slope***† ***p-value*** |  |  |
| **A^ғ^** | 11 | 11 | 0·03 |  | 13 | 11 | < 0·001 | 8 (1, 27) | 2 |
| **B^ғ^** | 10 | 10 | < 0·001 |  | 12 | 10 | < 0·001 | 6 (1, 25) | 2 |
| **C^ғ^** | 8 | 9 | < 0·001 |  | 11 | 9 | < 0·001 | 9 (2, 24) | 1 |
| **D^ғ^** | 9 | 10 | < 0·001 |  | 11 | 10 | < 0·001 | 8 (3, 18) | 2 |
| **E^ғ^** | 12 | 12 | 0·01 |  | 15 | 12 | 0·002 | 10 (2, 32) | 1 |
| **F^ғ^** | 9 | 10 | 0·05 |  | 14 | 10 | 0·001 | 17 (7, 42) | 1 |
| **G^ғ^** | 12 | 12 | < 0·001 |  | 14 | 12 | < 0·001 | 9 (2, 29) | 2 |
| **H^ғ^** | 17 | 19 | < 0·001 |  | 19 | 19 | < 0·001 | 17 (6, 53) | 1 |
| **I^ғ^** | 2 | 11 | 0·39 |  | 2 | 11 | 0·53 | 126 (93, 149) | 0 |
| **J^ғ^** | 15 | 21 | 0·19 |  | 19 | 21 | < 0·001 | 60 (30, 130) | 0 |
